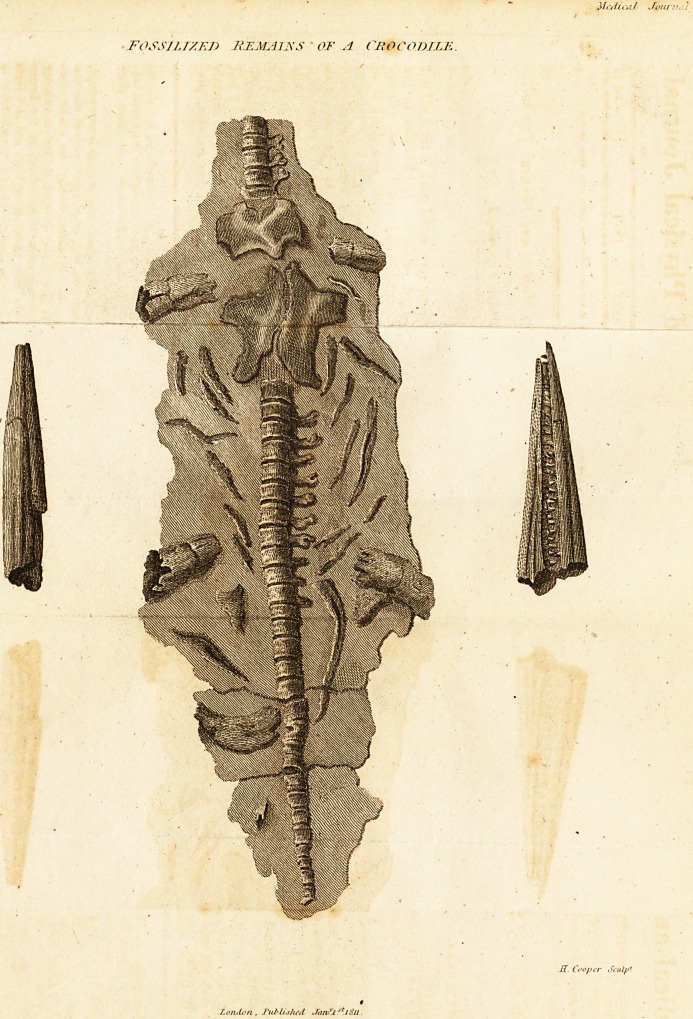# Remarks on Fossil Remains

**Published:** 1811-02

**Authors:** Joshua Brooks

**Affiliations:** Theatre of Anatomy, Blenheim-street


### Medical and Physical Journal.

VOL. XXV.] FEBRUARY, 1811. [NO. 144.

Printed for R. PHILLIPS, by E. Hemsted, Great New Street, Fetter Lane, London.

### *To the Editors of the Medical and Physical Journal.*

*(With an Engraving.)*

GENTLEMEN,

PERCEIVING that your very useful work is rendered not only valuable to the medical profession, but likewise to the naturalist; I beg to transmit to you for publication, a drawing of a curious extraneous fossil, should you think it of sufficient importance. To me this drawing is very interesting, inasmuch as it is the production of a young gentleman who studied anatomy at my Theatre.* It represents, with great spirit and correctness, the fossil remains of a young crocodile or alligator, an animal which perhaps was never indigenous in this island;† at least in any of the species at present existing.

That parts of apparently exotic animals are frequently found in this country, is well known to every naturalist: but there is a doubt whether these did actually belong to animals now only living near to the equator, or are remains, pre-

* This gentleman, Mr. Alfred Jukes, of Birmingham, has also favoured me with some fine representations of uncommon anatomical subjects, that have occurred in the dissections which have taken place within these last twelve months.

† That the climate of the British islands was, at some remote period, congenial to crocodiles, elephants, and other quadrupeds of the torrid zone, I cannot believe. An hypothesis founded on a wild conjecture of the position of this globe being changed, and with this change of position its temperature reduced, seems to be refuted by the beaver *(Castor fiber)* having formerly been an inhabitant here, and also from the fossil bones of the Elk *(Cervus elephas)* being frequently found in Ireland. Both these animals are now inhabitants of the frigid zone. A fair deduction from these premises would be, that in some early period, the climate of Britain was much colder than at present.

served by a process in natural chemistry, as evidence of the former existence of genera of animals, possibly, now extinct.

If the fossil remains discovered in this country did belong to races of animals now existing only within the tropics, two conjectures may be offered to account for their being found at this distance from their native soil, without having recourse to some violent change of climate to prove that they might be indigenous here.

The waters of the general deluge may have carried them from one quarter of the world to another, especially the amphibia, and being left in regions far from their natural climate, and subsiding in situations favourable to fossilization, their remains have been preserved to this time.

It is no improbable conjecture, that many of the bones of exotic animals dug up in various parts of this island, belonged to animals brought hither by the Romans. The passion this people had for collecting extraordinary creatures from every part of the world to which they had access, gives force to this opinion. That rare animal the *Camelopardalis Giraffa*, which we are now permitted to see in Europe only in dead specimens, was exhibited alive at Rome. The Roman Consul, Lucullus, brought hither the cherry, which being first planted in Kent, has taken the name of that county. The *Helix pomatia*, now so numerous in Surry and other parts, was imported by Sir Kenelm Digby. My friend, Mr. Winston, of Newman Street, presented me with the hoofs of a very large exotic deer, which were dragged from the bottom of the Thames,* along with the head of a lion; and I can point out the spot, where about thirty years ago the largest white bear (*Ursus maritimus*) ever seen in this country, is buried: but as it is under a public highway†, which has since been made, and as the ground is very much raised, it is almost impossible now to obtain the bones. Some centuries hence, perhaps the skeleton of this animal may be found in a mass of sulphate of lime, carbonate of lime, or other mineral;

* It is a curious fact that bones are so valuable an article of commerce, that men are employed incessantly in dragging the bottom of the river to obtain them. The animal oil being extracted from the bones thus obtained, the parts remaining, particularly of the shank bones, are rasped into hartshorn shavings, and are under this metamorphosis converted into jellies, &c. &c. for our splendid entertainments. The less delicate of these bones, or rather those which have an inconvenient shape for the action of the rasp, are, with a similar trading dexterity, made into ivory-black.

† At the entrance of Brook-street, a few yards from the middle of the New Road, and at a short distance from Tottenham Court, from which it bears west.

and and may then excite conjectures similar to those which now occupy our minds. If from any accident, in future times these bones should be discovered in a fossil state, the naturalist of that period will have his doubts and conjectures removed by this record, placed in a work which cannot fail to be consulted, in succeeding ages, by the physician and the man of science.

There is reason to believe, however, that many, possibly the greater portion of the fossil remains of large animals in these islands, and in many other parts of the globe, do not owe their deposition to either of the foregoing causes, having now no existing prototypes;* and that the bones of enormous size, frequently found very far beneath the surface, are the remains of extinct genera, once indigenous in the countries where these remains are discovered. Many facts are on record which give force and authority to this opinion. Some years since, the skeleton of an enormous sloth was found in Spain, of which I have a drawing. This skeleton measured twelve feet in length, and certainly was the remains of an animal incognitum; as the largest of the *Bradypus* genus (*Bradypus ursinus*) is about the ordinary size of the *Ursus arcticus*, or black bear. I have seen several fossil molar teeth of an animal incognitum (*carnivorous*), found on the coast of Essex; and nearly the size of those of the adult elephant. Similar productions may be seen in the British Museum; and in the private collections of many naturalists are preserved parts of unknown animals. The horns of an animal of the Beeve tribe have been found of an enormous magnitude, belonging to a creature of whose present existence we have no knowledge. The most extraordinary among these remains of former times is the claw of a quadruped (probably rapacious) of a size exceeding all belief, were it not now to be seen in Mr. Bullock's Museum.† Even human bones

* We must not positively decide that an extinction of these races of animals has happened. Perhaps a farther exploration of this earth may bring us acquainted with some of them in a living state. Many creatures have been brought into Europe, and particularly into England, within a few years, of whose existence we had not the most distant idea. The *Elephas americanus* may yet live; and the rara avis of Juvenal* is no longer a wonder, now we have found the *Anas atrata*. The discovery of the *Macropus gigantea*, the *Ornithoryncus paradoxus*, and the *Syren lacertina*, ought not to make us credulous, but they may make us doubt what forms of being range in unexplored forests, or swim in unknown seas.

† The Museum of Mr. Bullock, in Piccadilly, has in it many rare and curious specimens of nature, collected at a great expence, and with an

* "*Rara avis in terris, nigroque simillima cygno.*" Sat. vi.

 ardour have been found of a length and dimensions, which almost countenance the opinion that a race of giants once existed on this earth. Crania have likewise been discovered, sunk deep in the earth, where they must have lain many centuries, varying so materially from the form of the head in the present race of men, as to baffle conjecture and bewilder investigation. In the valuable collection of Mr. Heavyside, is a human skull, which was found 200 feet below the surface of the soil, (the workmen who were sinking a well in which this skull was found, supposed at this depth they were digging maiden earth,) remarkable for its length, and the form of the *ossa nasalia.** Another cranium, which was presented to me by Mr. Nicholls of Margarett Street, is as singular for its shortness, and for peculiarities in its general conformation.†

The most extraordinary perhaps, certainly the most noticed of these antient remains, are the fragments, and even the whole of the boney structure of an animal which has had given to it the appellation of *mammoth*. The bones of this creature have generally been found in Russia, Siberia, and North America; and some in England. It is but a few years since Mr. Peele exhibited the skeleton of one of three which were discovered in a swamp in the province of Pennsylvania; and the evidence for its having been indigenous in the northern climates of the old world, is very satisfactory. Not many years ago an unusually hot summer dissolved the snow so much in one of the northern provinces of Russia, as to lay open an immense cavern, in which were several mammoths entire.‡ But unfortunately, as soon as the bodies of these animals were thawed, bears, wolves, &c. regaling upon the novel repast, left little more than the bones. Some portion, however, of muscle and integument were brought away; and in the splendid Museum of the Royal College of Surardour in the study of natural history, which reflects great credit on the ingenious proprietor. Editor.

* If these facts are not stated with precision, Mr. Heavyside will, possibly, have the goodness to correct them.

† This skull was found on removing the rubbish from beneath the foundation of a building in the parish of Marylebone, known to have stood 400 years.

‡ A complete mammoth was found in a state of perfect preservation, on the coast of the frozen ocean, by Schoumachoff, a Tongoose chief, in the autumn of 1799, in the midst of a rock of ice. A detailed history of this fact may be seen in the Phil. Mag. 29. 141. The largest os femoris of the mammoth ever seen, was found in Cheshire in a fossil state, now at Mr. Bullock's museum.

geons geons of London, by the favour of Sir James Earl, I examined a portion of cutis clothed with fine soft fur, obtained from one of these tremendous creatures.

The extraneous fossil, represented in the annexed engraving, and discovered in a stone quarry, thirty feet below the surface, at Wilmcote, near Stratford upon Avon, in the spring of 1810, undoubtedly belongs to the *Lacerta* genus; but whether it is an individual of any of the known existing species is not to be fully determined. The disagreement both in the disposition and form of the teeth in this fossil specimen with its congenors, lead rather to a conjecture that it may be an individual of an extinct species,* which was found to exist in the climate of the British islands.

None of the known species of crocodile will live many years in this country, and indeed, like other species of the amphibia, will often not feed at all. The longest period a crocodile has been kept alive here was fourteen years. This animal was in the possession of my father, and when it died was presented to Mr. John Hunter, and may probably have been the individual in which he noticed the singular fact of chyle in the lacteals.

I am, Gentlemen,
Your's, &c. &c.
JOSHUA BROOKS.

*Theatre of Anatomy, Blenheim-street,*
*January* 7, 1811.

---

### *To the Editors of the Medical and Physical Journal.*

### *Cases of Scarlatina Anginosa, treated by* Mr. R. HAMILTON *and his* NEPHEW.

GENTLEMEN,

FINDING some remarks and cases of this formidable and fatal disease, in your last Number, by Mr. Goodwin, who

---

* The obscurity which necessarily accompanies fossillized substances must prevent positive decision. Characteristic marks of species may be so altered, obliterated, or blended with carbonate of lime, &c. as no longer to deserve that denomination. The disagreement in our specimen with the existing species, is of a kind not likely to be produced by the process of fossillization. As far as can he determined from the drawing, the correctness of which is not to be doubted, the teeth do not agree, either in form or arrangement, with the corresponding animals of the genus Lacerta. This fossil is five feet nine inches long.

## Figures and Tables

**Figure f1:**